# Near-Infrared Activation of Sensory Rhodopsin II Mediated by NIR-to-Blue Upconversion Nanoparticles

**DOI:** 10.3389/fmolb.2021.782688

**Published:** 2022-01-19

**Authors:** Momo Yaguchi, Xiaodan Jia, Ramona Schlesinger, Xiue Jiang, Kenichi Ataka, Joachim Heberle

**Affiliations:** ^1^ Experimental Molecular Biophysics, Department of Physics, Freie Universität Berlin, Berlin, Germany; ^2^ State Key Laboratory of Electroanalytical Chemistry, Changchun Institute of Applied Chemistry, Chinese Academy of Science, Changchun, China; ^3^ Genetic Biophysics, Department of Physics, Freie Universität Berlin, Berlin, Germany

**Keywords:** microbial rhodopsins, FTIR spectroscopy, photocycle, near-infrared light, lanthanide-doped upconversion nanoparticles, optogenetics

## Abstract

Direct optical activation of microbial rhodopsins in deep biological tissue suffers from ineffective light delivery because visible light is strongly scattered and absorbed. NIR light has deeper tissue penetration, but NIR-activation requires a transducer that converts NIR light into visible light in proximity to proteins of interest. Lanthanide-doped upconversion nanoparticles (UCNPs) are ideal transducer as they absorb near-infrared (NIR) light and emit visible light. Therefore, UCNP-assisted excitation of microbial rhodopsins with NIR light has been intensively studied by electrophysiology technique. While electrophysiology is a powerful method to test the functional performance of microbial rhodopsins, conformational changes associated with the NIR light illumination in the presence of UCNPs remain poorly understood. Since UCNPs have generally multiple emission peaks at different wavelengths, it is important to reveal if UCNP-generated visible light induces similar structural changes of microbial rhodopsins as conventional visible light illumination does. Here, we synthesize the lanthanide-doped UCNPs that convert NIR light to blue light. Using these NIR-to-blue UCNPs, we monitor the NIR-triggered conformational changes in sensory rhodopsin II from *Natronomonas pharaonis* (*Np*SRII), blue light-sensitive microbial rhodospsin, by FTIR spectroscopy. FTIR difference spectrum of *Np*SRII was recorded under two different excitation conditions: (ⅰ) with conventional blue light, (ⅱ) with UCNP-generated blue light upon NIR excitation. Both spectra display similar spectral features characteristic of the long-lived M photointermediate state during the photocycle of *Np*SRII. This study demonstrates that NIR-activation of *Np*SRII mediated by UCNPs takes place in a similar way to direct blue light activation of *Np*SRII.

## Introduction

Microbial rhodopsins are retinal-containing membrane proteins pivotal in cellular behaviors. Upon light illumination, the retinal chromophore that is covalently bound to the protein via a protonated Schiff base undergoes photoisomerization, followed by a cyclic reaction of conformational changes in the protein, known as photocycle, which eventually leads to a cellular response. Making good use of the sensitivity to visible light, microbial rhodopsins present powerful tools in optogenetics, a method that exploits light to control cellular responses. Limited penetration depth of visible light in biological tissue (Yaroslavsky et al., 2002; Bashkatov et al., 2005; Knöpfel et al., 2010; Lin, 2011; Häusser, 2014), however, is challenging and ways to effectively deliver light to the target protein in deep tissue must be found. While implantations of optical fibers (Aravanis et al., 2007) and miniature light-emitting diode devices (Kim et al., 2013) into deep-tissue brain succeeded in direct protein activation by visible light, tissue damage and physical restriction of the subject remain principal limitations. Genetically engineered red light-active optogenetic proteins (Lin et al., 2013; Chuong et al., 2014; Klapoetke et al., 2014; Marshel et al., 2019) have gained deeper tissue penetration, but effective light delivery still relies on invasive approaches.

The limitations can be resolved by applying near-infrared (NIR) light that reaches deeper in biological tissue than visible light and causes minimal photodamage (Zonios et al., 2001; Yaroslavsky et al., 2002; Bashkatov et al., 2005). However, the current lack of NIR-sensitive microbial rhodopsins requires a local optical transducer that can bridge the gap in wavelengths between NIR and visible radiation. For this purpose, lanthanide-doped upconversion nanoparticles (UCNPs) benefit wireless optogenetics because they convert low-energy NIR radiation into high-energy visible light (Auzel, 2004; Wang and Liu, 2009; Chen et al., 2014; Zhou J. et al., 2015). These lanthanide-doped UCNPs are commonly composed of inorganic host nanocrystals co-doped with lanthanide ions that have ladder-like energy levels. Upon NIR light excitation, the incident photons are absorbed by lanthanide ions (sensitizer), and the harvested energy is transferred to the other lanthanide ions (activator) that emit upconverted visible light. The completely shielded 4f orbitals of lanthanide ions account for the long-lived excited states as well as the narrow-band light emission, highlighting the unique optical properties of lanthanide-doped UCNPs. Additionally, they are low cytotoxic and biocompatible nanocrystals (Haase and Schäfer, 2011; Zhou B. et al., 2015; Hososhima et al., 2015), which have promoted the application of lanthanide-doped UCNPs to wireless optogenetics with NIR light.

UCNP-assisted optogenetics enables less-invasive, deep-tissue accessible, less tissue-damaging, and physically unrestricted activation of proteins with NIR light. Previous literature reports have demonstrated the feasibility of this strategy (Hososhima et al., 2015; Shah et al., 2015; Ai et al., 2017; Lin et al., 2017; Pliss et al., 2017; Wang et al., 2017; Yadav et al., 2017; Chen et al., 2018; Lin et al., 2018; Ma et al., 2019; Miyazaki et al., 2019), most commonly using electrophysiological technique. While electrophysiology is a powerful method to test the functional response of the target protein, mechanistic details on the photocycle of the target protein when activated with NIR radiation remain an open question. Unlike conventional light sources, UCNP-protein interactions may affect the photoreaction of the target protein. Given that lanthanide-doped UCNPs have generally multiple emission peaks at different wavelengths, such mechanistic insight helps elucidate if the photocycle of proteins activated with NIR light in the presence of the UCNPs takes place in a similar way to that of proteins activated with visible light. Herein, we investigate how the activation of sensory rhodopsin II from *Natronomonas pharaonis* (*Np*SRII) takes place with NIR light in the presence of lanthanide-doped UCNPs that convert NIR light into blue light. To probe the conformational changes of *Np*SRII elicited by light illumination, we employ FTIR spectroscopy in an attenuated total reflection (ATR) configuration and record FTIR difference spectrum between the resting and active states. The acquired FTIR difference spectra resolve key photointermediate states prevalent during the photocycle, which is compared with spectra recorded under blue light illumination. *Np*SRII is blue light sensitive microbial rhodopsin and acts as outward directed proton pump in the absence of its cognate transducer protein (Sudo et al., 2001). Because *Np*SRII undergoes much slower photocycle than bacteriorhodopsin (bR), it provides an ideal platform for tracking the photointermediate states under photostationary conditions. The UV/Vis absorption spectrum of *Np*SRII is very similar to *Cr*ChR2 (channelrhodopsin-2 from *Chlamydomonas reinhardtii*) which is the most prominent optogenetic tool. Lanthanide-doped UCNPs are synthesized by co-doping two different lanthanide ions, Yb^3+^ and Tm^3+^, within the inorganic framework of NaYF_4_, and emit blue light upon irradiation with 980 nm light. Using this lanthanide-doped UCNP, *Np*SRII can be indirectly activated by NIR light. The acquired FTIR difference spectrum reveals that the M state is mostly accumulated under photostationary conditions, corroborating that the photocycle of *Np*SRII under NIR light illumination is analogous to that under blue light illumination.

## Materials and Methods

### Materials

YCl_3_.·6H_2_O (99.99%, metal basis), YbCl_3_.·6H_2_O (99.99%, metal basis), TmCl_3_·6H_2_O (99.99%, metal basis), NH_4_, NaOH and oleic acid were purchased from Aladdin Reagent Co., Ltd. 1-Octadecene, methanol and cyclohexane were purchased from Shanghai Macklin Biochemical Co., Ltd. Cyclohexane (99.5%) and tris(hydroxymethyl)aminomethane (>99.8%) were purchased from Sigma Aldrich. NaCl (≥99.5%) was purchased from Carl Roth. DDM (>99%, *n*-dodecyl-β-d-maltoside) was purchased from Glycon Biochemicals. MES (2-(N-morpholinyl)ethanesulfonic acid) were purchased from Sigma Aldrich. All chemicals were used without additional purification. Millipore Type 1 water (18.2 MΩ·cm) was used throughout the study.

### Methods

NaYF_4_:20%Yb 0.5%Tm nanoparticles were synthesized according to the previously reported solvothermal methods (Gong et al., 2019; Zhang et al., 2019). Briefly, 2 mmol YCl_3_.6H_2_O, YbCl_3_·6H_2_O, and TmCl_3_.6H_2_O in a ratio of 79.5: 20: 0.5 were added to the mixture of 15 ml oleic acid and 30 ml 1-octadecene in a 100 ml flask reactor on a Schlenk line. After the flask was purged with N_2_, the mixture was then heated to 160°C and kept for about 1 h with magnetic stirring to form a clear yellow solution. After the reaction system was cooled down to room temperature, 10 ml of methanol containing 5 mmol NaOH and 8 mmol NH_4_F was slowly added to the three-necked flask. The system was kept at 50°C for 30 min, then heated to 70°C and kept for another 30 min to vaporize most of the methanol. Thereafter, the system was heated to 100°C and cycled three times between vacuum and nitrogen atmosphere to remove residual methanol, water and oxygen. Subsequently, the solution was quickly heated up to 300°C and maintained at this temperature for 1.5 h under the protection of nitrogen atmosphere. After the system was cooled down naturally, the nanoparticles were collected by centrifugation and washed three times with ethanol.

The morphology of the synthesized lanthanide-doped UCNPs was observed by transmission electron microscope (H-600 electron microscope, Hitachi). Energy-dispersive X-ray elemental mapping images were obtained by a FEI TECNAI G2 high-resolution transmission electron microscope operating with a field-emission gun operating at 200 kV. Powder X-ray diffraction (XRD) spectra were gathered using a D8 ADVANCE X-ray diffractometer (Bruker, Cu Kα radiation, λ = 1.5418 Å). Upconversion emission spectra were acquired on a Cary Eclipse Fluorescence Spectrometer (Agilent Technologies) externally equipped with a 980 nm continuous wave laser (Changchun New Industries Optoelectronics Tech Co.,Ltd.).

*Np*SRII with a histidine tag at the C-terminus was expressed in *Escherichia coli* BL21 (DE3) RP cells and purified on a Ni-NTA column essentially as described for *Hs*SRI-HtrI (Orekhov et al., 2017). *Np*SRII sample (5.2 mg mL^−1^) was washed four times with detergent-free buffer (50 mM NaCl, 5 mM TRIS, pH 8.0) in a concentrator (Amicon^®^ Ultra-0.5 Centrifugal Filter Devices, Merck Millipore) using a Hettich Mikro-22R centrifuge at 13540xg for 20 min. UV-Vis absorption spectrum was recorded with a Shimadzu UV2600 UV-VIS spectrophotometer. FTIR measurements were conducted using a Bruker Vertex 70 FTIR spectrometer equipped with a HgCdTe (MCT) detector and an attenuated total reflection (ATR) accessory. In order to check the dryness of the protein film, FTIR spectrum of drop-casted 8 μL of *Np*SRII on the ATR Si surface was acquired after 0 and 30 min with a spectral resolution of 4 cm^−1^ with 128 co-added scans.

The emission from a 980 nm continuous wave NIR laser (Changchun New Industries Optoelectronics Tech Co., Ltd.) was applied for excitation of the lanthanide-doped UCNPs. All FTIR spectroscopic measurements were carried out on a Bruker Vertex 70 FTIR spectrometer equipped with a HgCdTe (MCT) detector and an attenuated total reflection (ATR) accessory. Spectra were acquired with a spectral resolution of 4 cm^−1^ with 128 co-added scans. This procedure was repeated four times and the spectra were averaged. For light-induced FTIR difference spectroscopic measurements with visible and UV light, *Np*SRII was illuminated with a light-emitting diode (LED) with emission maximum at 495 nm (Luxeon Star LEDs), 475 nm (Roschwege) and 365 nm (Roschwege). The bandwidths of 495 nm, 475 nm and 365 nm LEDs are 25, 20 and 16 nm, respectively. A single-beam spectrum collected without light illumination was used as the background spectrum. All experiments were performed at room temperature.

## Results and Discussion

**FIGURE 1:**
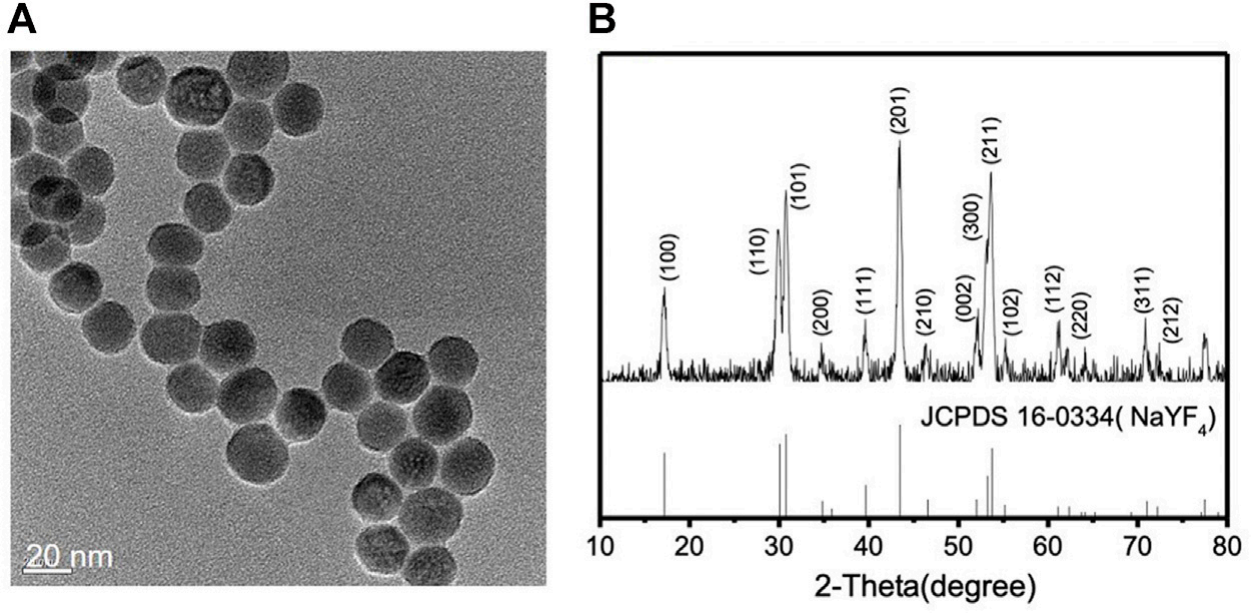
**(A)** TEM image and **(B)** XRD patterns of the lanthanide-doped UCNPs (NaYF_4_: 20 mol% Yb^3+^/0.5 mol% Tm^3+^), top, and the β-NaYF_4_, bottom.

Lanthanide-doped NIR-to-blue UCNPs (NaYF_4_: 20 mol% Yb^3+^/0.5 mol% Tm^3+^) were synthesized by previously reported solvothermal methods (Gong et al., 2019; Zhang et al., 2019). Two different lanthanide ions, Yb^3+^ and Tm^3+^, are co-doped within the inorganic framework of hexagonal phase NaYF_4_ nanocrystals. Yb^3+^ ions have only one excited 4f level that effectively absorbs the energy from the 980 nm NIR light due to the ^2^F_7/2_ → ^2^F_5/2_ transition. Tm^3+^ ions are chosen because it is known that the energy transfer from Yb^3+^ to Tm^3+^ is efficient and they emit blue light (Wang and Liu, 2009; Haase and Schäfer, 2011). The synthesized lanthanide-doped UCNPs were characterized by transmission electron microscopy (TEM), powder X-ray diffraction (XRD), energy dispersive X-ray spectroscopy (EDX), and FTIR spectroscopy. The spherical lanthanide-doped UCNPs are monodispersed and uniform in size as shown in the TEM image, Figure 1A. From a detailed size distribution analysis, the average particle size was found to be 21.63 nm with a standard deviation of 2.5 nm (Supplementary Figure S1). Figure 1B reveals that the XRD patterns of the lanthanide-doped UCNPs correspond to the standard XRD patterns of *β*-NaYF_4_ (JCPDS 16–0334). EDX analysis of the lanthanide-doped UCNPs confirms the presence of all components (Supplementary Figure S2). The absence of the C=O stretching vibration at 1,706 cm^−1^ in the FTIR spectrum of the lanthanide-doped UCNPs suggests that the surface of the lanthanide-doped UCNPs are capped by oleate species originated from oleic acid used in the synthesis (Supplementary Figure S3), consistent with previous literature reports (Bogdan et al., 2011; Chen et al., 2012; Liang et al., 2018; Ao et al., 2019; Thanasekaran et al., 2019).

**FIGURE 2:**
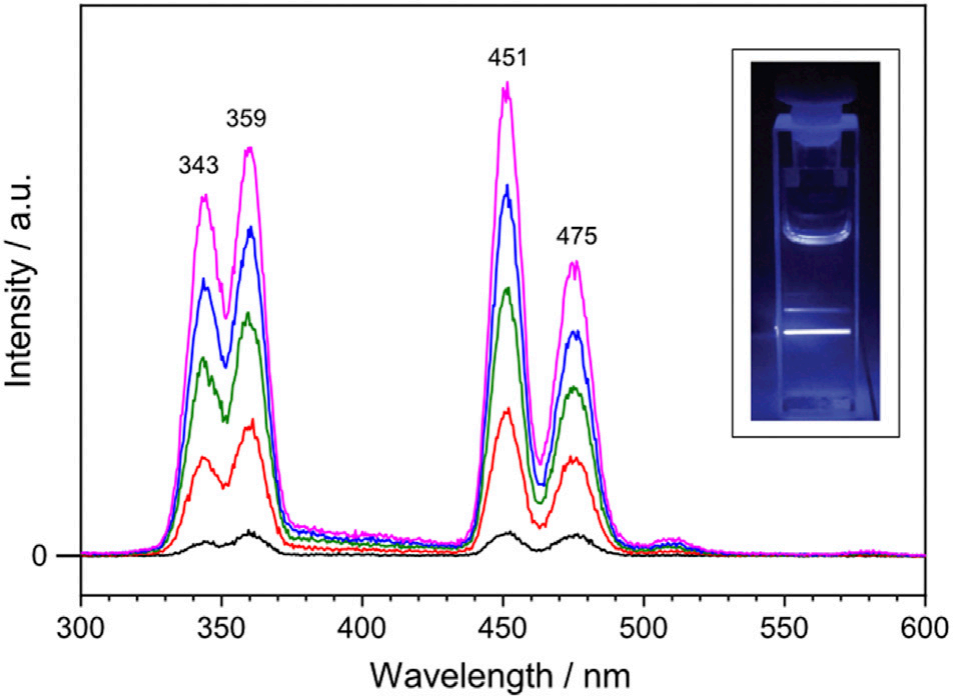
Upconverted photoluminescence spectrum of the lanthanide-doped UCNPs excited with 980 nm continuous wave laser at varied output power: 0.4, 0.8, 1.2, 1.6 and 2.0 W colored in black, red, green, blue and magenta, respectively. The lanthanide-doped UCNPs are dissolved in cyclohexane ([UCNP] = 1 mg mL^−1^). Inset shows a photograph of the blue light emission generated by the lanthanide-doped UCNPs upon NIR excitation at 1.0 W.

To investigate the optical properties of the synthesized lanthanide-doped UCNPs, upconverted photoluminescence spectra were recorded. The lanthanide-doped UCNPs were dissolved in cyclohexane ([UCNP] = 1 mg mL^−1^) and the solution was excited with a continuous wave 980 nm diode laser. Figure 2 shows the upconverted photoluminescence spectrum of the lanthanide-doped UCNPs taken with varied 980 nm NIR excitation output power: 0.4, 0.8, 1.2, 1.6 and 2.0 W colored in black, red, green, blue and magenta, respectively. Four emission bands characteristic for Tm^3+^ ions are observed at 343, 359, 451 and 475 nm, corresponding to the decay of ^1^I_6_ → ^3^F_4_, ^1^D_2_ → ^3^H_6_, ^1^D_2_ → ^3^F_4_, and ^1^G_4_ → ^3^H_6_, respectively. Among them, two emission bands of Tm^3+^ in the blue region, 451 and 475 nm, reveal that the synthesized lanthanide-doped UCNPs absorb 980 nm NIR light and emit the upconverted blue light, which was visible to the naked eye (Figure 2, inset). Within the applied power range, the output power of the NIR laser displays a linear correlation with the intensity of the emission bands at 451 and 475 nm (Supplementary Figure S4).

**FIGURE 3:**
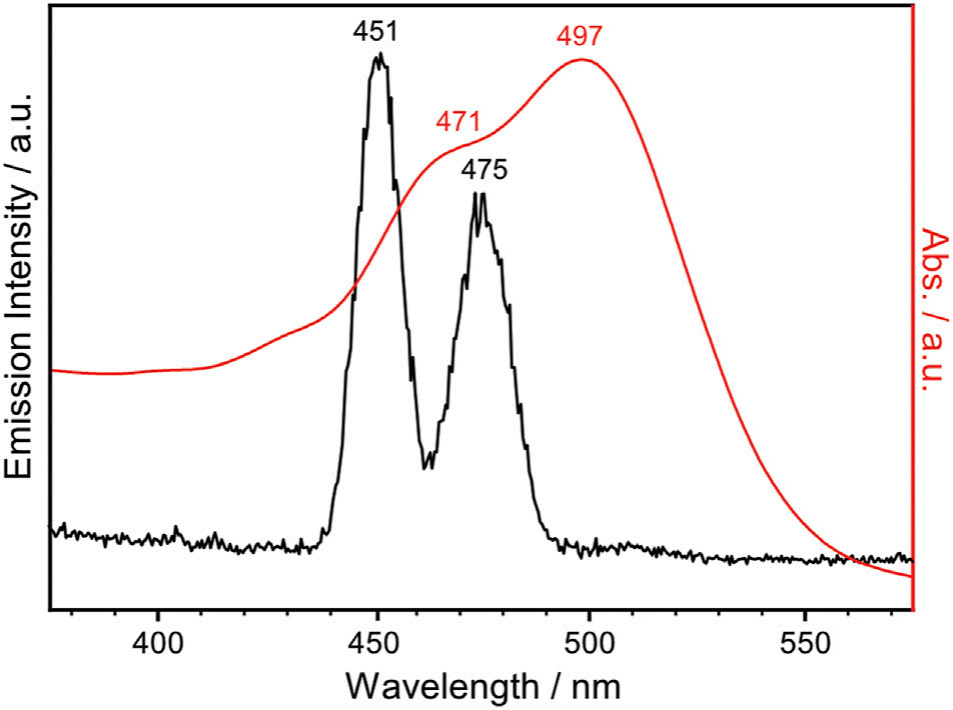
UV-Vis absorption spectrum of *Np*SRII (red trace), overlaid with the emission spectrum of the lanthanide-doped UCNPs in cyclohexane ([UCNP] = 1 mg mL^−1^) excited with 980 nm continuous wave laser at 0.6 W (black trace).

*Np*SRII is a heptahelical transmembrane protein containing a retinal chromophore, whose photoisomerization is triggered by blue light illumination. The photocycle of *Np*SRII contains several sequential photointermediate states that are spectrally distinctive (Chizhov et al., 1998): from the ground state to K, L, M, N and O intermediate state with absorption maximum of 500, 510, 495, 400, 485 and 535 nm, respectively. While *Np*SRII acts as phototaxis receptor that mediates blue light avoidance in the presence of its cognate transducer protein (HtrII), it also acts as an outward directed proton pump in the absence of HtrII (Sudo et al., 2001). Prolonged photocycle of *Np*SRII similar to bR is suitable for probing the photointermediate state during the photocycle with FTIR spectroscopy. Crystal structure of *Np*SRII (Luecke et al., 2001; Gordeliy et al., 2002) and spectroscopic studies on the photocycle of *Np*SRII under blue light illumination (Hirayama et al., 1992; Engelhard et al., 1996; Chizhov et al., 1998; Furutani et al., 2002; Hein et al., 2003; Furutani et al., 2004; Iwamoto et al., 2004; Bergo et al., 2005; Mironova et al., 2005; Jiang et al., 2008; Jiang et al., 2010; Tateishi et al., 2011; Mohrmann et al., 2016; Pfitzner et al., 2018) have been previously reported. Given this literature precedent, we record the FTIR difference spectrum of *Np*SRII under NIR light illumination. *Np*SRII proteins were produced and purified as previously described (Hohenfeld et al., 1999; Orekhov et al., 2017). The *Np*SRII film for FTIR spectroscopic measurements was prepared by drop-casting 8 μL of purified *Np*SRII on the silicon ATR crystal. Strong water bands at around 3,400 and 1,600 cm^−1^ in the FTIR spectrum taken right after drop-casting gradually declined over time, and no significant changes were probed in their intensities after 30 min of air-drying. This decline in water bands is accompanied by the rise in methyl bands (3,000–2,800 cm^−1^), amide I band (1,654 cm^−1^) and amide II band (1,545 cm^−1^), resulting in the final amide I band intensity of around 0.5 (Supplementary Figure S5). In Figure 3, the UV-Vis absorption spectrum of the *Np*SRII film, colored in red, is overlaid with the emission spectrum of the lanthanide-doped UCNPs, colored in black. The emission bands at 451 and 475 nm of the lanthanide-doped UCNPs overlap with the absorption maxima of *Np*SRII centered at 471 and 497 nm, indicating that the blue light generated by lanthanide-doped UCNPs is capable of activating *Np*SRII. In addition, it is worthwhile to note that photonic excitation at shorter wavelengths is advantageous to avoid photoactivation of red-shifted intermediate states.

**FIGURE 4:**
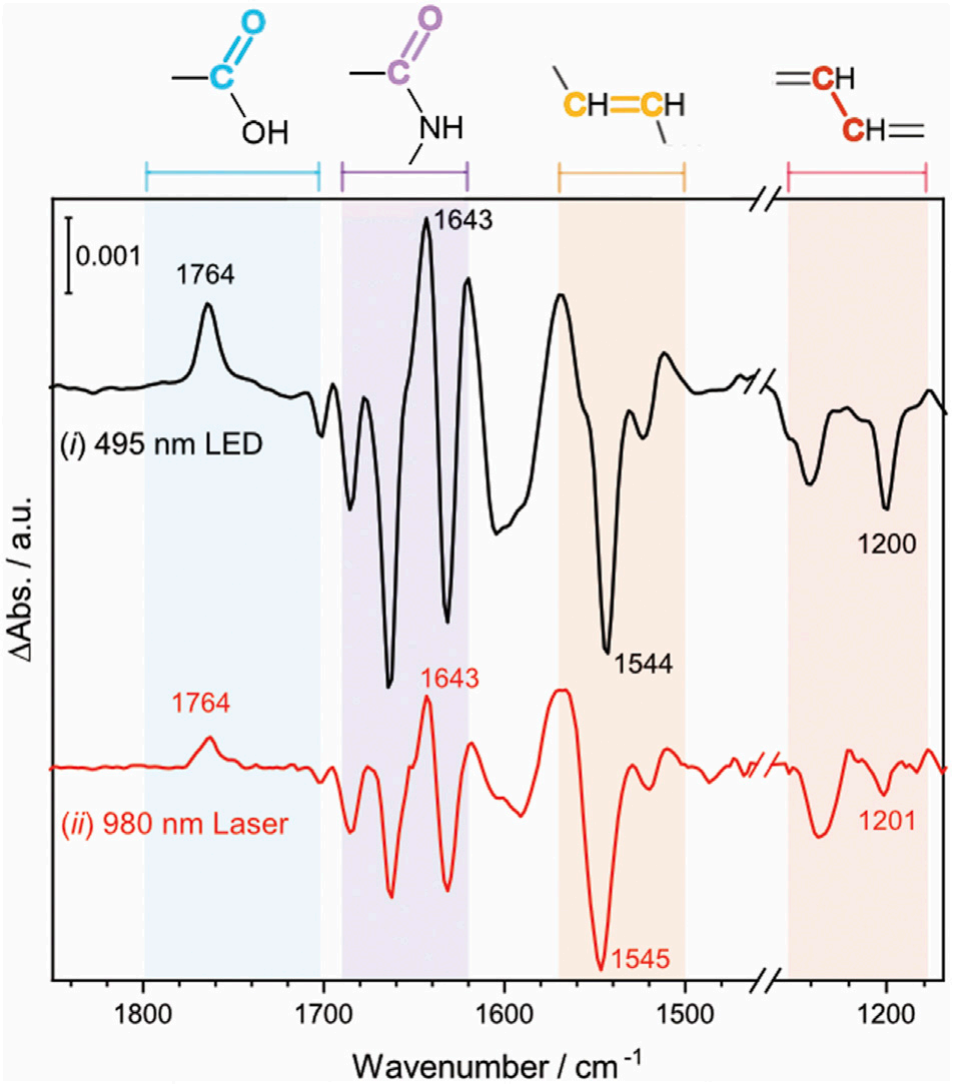
Light-induced FTIR difference spectrum of *Np*SRII acquired in two different conditions (ⅰ) *Np*SRII was directly illuminated with 495 nm blue LED light (ⅱ) *Np*SRII was indirectly illuminated with blue light generated by the lanthanide-doped UCNPs upon NIR excitation at 0.6 W, black and red traces, respectively. The colored areas refer to the frequency ranges of the C=O stretching vibration of carboxylic amino acid side chains (1,790–1,700 cm^−1^, pale blue), the amide I vibration of the peptide bonds (1,690–1,620 cm^−1^, pale purple), the C=C (1,570–1,500 cm^−1^, pale orange) and C-C stretching vibrations (1,240–1,160 cm^−1^, pale red) of the retinal chromophore.

To investigate the effect of the UCNP-generated blue light on the activation of *Np*SRII, FTIR difference spectroscopy measurements were carried out on the same *Np*SRII film under two different conditions: (ⅰ) direct excitation of *Np*SRII with 495 nm blue LED light, colored in black, (ⅱ) indirect excitation of *Np*SRII with blue light generated by the lanthanide-doped UCNPs upon NIR irradiation at 0.6 W, colored in red (Figure 4). Powdery lanthanide-doped UCNPs were manually cast over the entire surface of the *Np*SRII film to obtain a fairly homogeneous distribution of the UCNPs on the protein. For both conditions, the background spectrum was recorded without light illumination, and therefore the negative and positive bands in the spectrum correspond to the vibrations in the dark state and of photointermediate states prevalent during the photocycle, respectively. The spectral features observed in both spectra reveal striking similarities, inferring that blue light generated from the lanthanide-doped UCNPs upon NIR excitation activates *Np*SRII in a similar way as the conventional blue light does. It is known for *Np*SRII that under photostationary conditions, a mixture of M and O photointermediate states is formed and the relative abundance of each of these states is highly dependent on experimental conditions (Chizhov et al., 1998; Klare et al., 2002; Furutani et al., 2004; Iwamoto et al., 2004; Jiang et al., 2010). Four specific frequency ranges of the spectra are marked in a different color: the C=O stretching vibration of carboxylic amino acid side chains (1,790–1,700 cm^−1^, pale blue), the amide I vibration of the peptide bonds (1,690–1,620 cm^−1^, pale purple), the C=C (1,570–1,500 cm^−1^, pale orange) and C-C stretching vibrations (1,240–1,160 cm^−1^, pale red) of the retinal chromophore. The appearance of a band at 1,764 cm^−1^, assigned to the C=O stretching vibration of Asp75, indicates proton transfer from the retinal Schiff base to the counterion Asp75. Negative bands at 1,544 cm^−1^ and 1,200 cm^−1^ are indicative for the retinal in all-*trans* configuration. These spectral features, together with a positive band at 1,643 cm^−1^ in the amide I region, suggest that the M photointermediate state is prevalent during the photocycle in this study, carried out with *Np*SRII in detergent under weak alkaline conditions, which is consistent with previous literature reports (Engelhard et al., 1996; Jiang et al., 2010).

**FIGURE 5:**
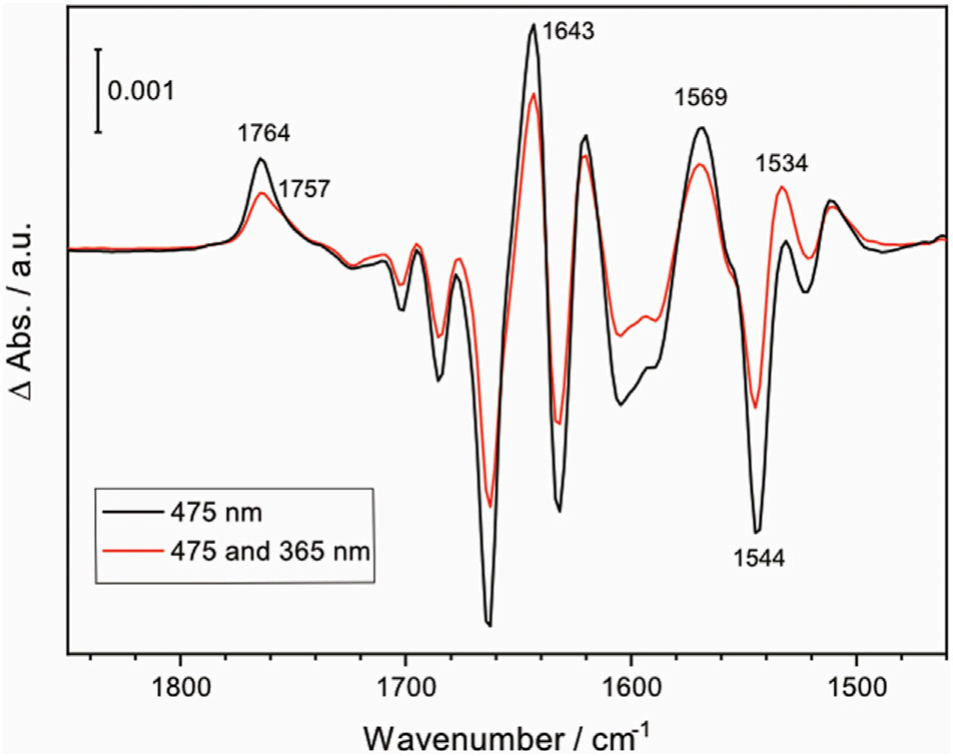
FTIR difference spectrum of *Np*SRII illuminated with 475 nm LED light, and with both 475 nm and 365 nm LED lights (black and red, respectively).

While direct activation of *Np*SRII is conventionally carried out with narrow-band blue light, the synthesized lanthanide-doped UCNPs emit not only blue light but also UV light. To examine if UV illumination has an effect on the photoreaction of *Np*SRII, FTIR difference spectra were recorded under both blue and UV light illumination, thereby simulating the experiments of UCNP-mediated NIR activation of *Np*SRII. Figure 5 shows the light-induced FTIR difference spectra of *Np*SRII recorded under 475 nm illumination only (black trace), and with simultaneous illumination of 475 nm and 365 nm light (red trace). While general spectral features in both spectra resemble well regardless of the different wavelengths used for photoexcitation, the difference spectrum taken together with UV light exhibits weaker band intensities compared to the one taken with only blue light. This result apparently demonstrates quenching of the M state by UV light (Balashov et al., 2000). Furthermore, characteristic bands of the M state (1,764 cm^−1^, 1,643 cm^−1^, 1,569 cm^−1^, and 1,544 cm^−1^) display reduced intensities. Appearance of a shoulder at 1,757 cm^−1^ and the higher intensity of the ethylenic mode of retinal at 1,534 cm^−1^ (Furutani et al., 2004) indicate that the photostationary state is shifted towards the O state by UV light illumination. Time-resolved FTIR studies are underway to gain further insight into the kinetics of NIR-activated *Np*SRII in the presence of the UCNPs.

## Conclusion

In conclusion, we have synthesized the lanthanide-doped UCNPs (NaYF_4_: 20 mol% Yb^3+^/0.5 mol% Tm^3+^) that absorb 980 nm NIR light and emit UV and blue light. With these UCNPs, we have conducted the FTIR spectroscopic measurements on the NIR activation of *Np*SRII to probe the conformational changes in the protein. The FTIR difference spectrum of *Np*SRII recorded under NIR illumination in the presence of the lanthanide-doped UCNPs exhibits spectral features characteristic of the M state during the photocycle, consistent with the one recorded under blue light illumination in the absence of UCNPs. We show that UCNP-generated UV light affects the photocycle of *Np*SRII by quenching the M photointermediate states. This work provides the first spectroscopic insight into the photoreaction of *Np*SRII activated with NIR light in the presence of UCNPs, and, thus contributes to further establish UCNP-assisted optogenetics with NIR light of bulk biological materials such as tissue and organs.

## Data Availability

The original contributions presented in the study are included in the article/Supplementary Material, further inquiries can be directed to the corresponding authors.
