# Supplementary material for: Near-Infrared Activation of Sensory Rhodopsin II Mediated by NIR-to-Blue Upconversion Nanoparticles
Source: Front Mol Biosci. 2022 Jan 19;8:782688. doi: 10.3389/fmolb.2021.782688 (PMC8892918; doi:10.3389/fmolb.2021.782688)
Supplement: Supplementary file 1 [file DataSheet1.docx]

Supplementary Material

## Supplementary Figures


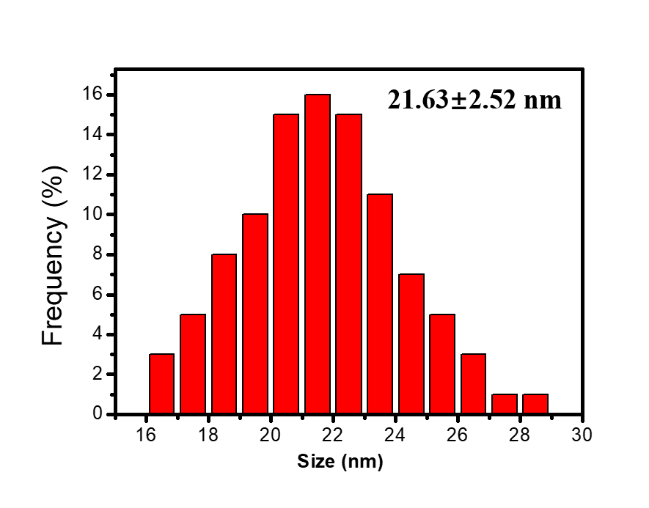


**Supplementary Figure 1.** Size distribution chart for the synthesized UCNPs (NaYF_4_: Yb, Tm nanocrystals). The average particle size was 21.63 nm with a standard deviation of 2.52 nm.

**
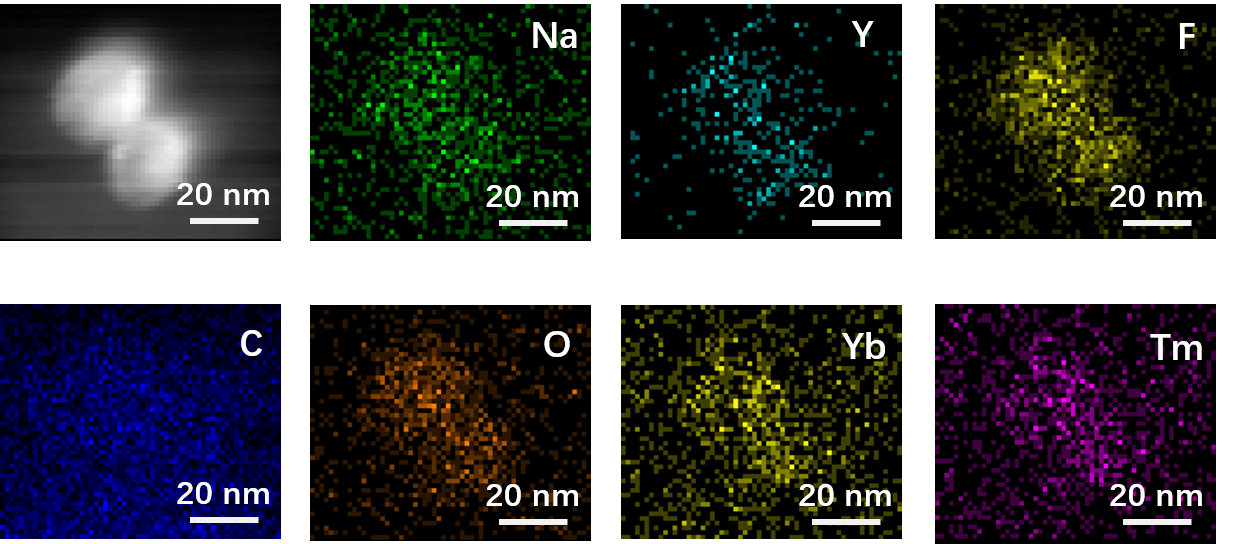
**

**Supplementary Figure 2.** Dark-field STEM image (top, left) and EDX elemental mapping of the synthesized UCNPs (NaYF_4_: Yb, Tm nanocrystals).


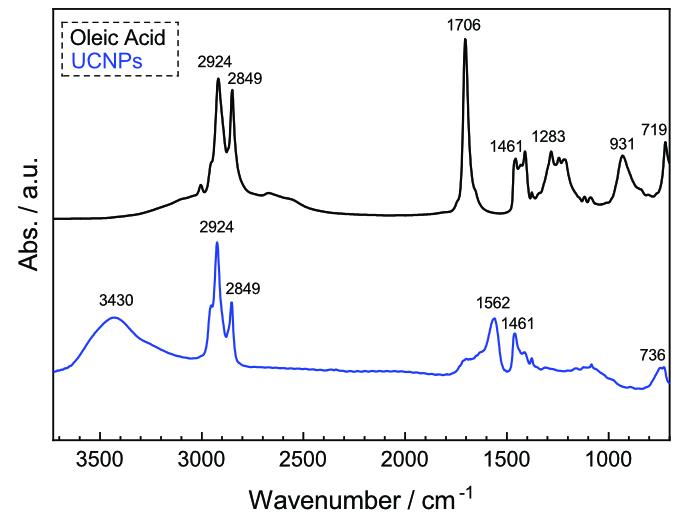


**Supplementary Figure 3.** FTIR spectra of the synthesized UCNPs (bottom, blue) and pure oleic acid (top, black). The bands at 2924 and 2849 cm^-1^ are assigned to the asymmetric and symmetric stretching vibration of methylene in the long alkyl chain of the oleic acid molecules, respectively. The band at 1706 cm^-1^ is attributed to the C=O stretching vibration, which stands out in oleic acid but not in the UCNPs, suggesting the oleate-capped surface of the UCNPs.


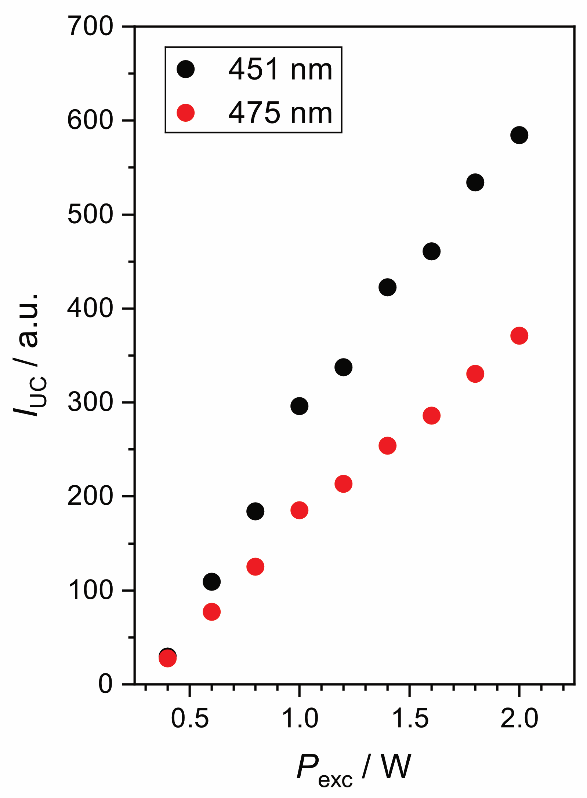


**Supplementary Figure 4.** Upconverted emission band intensity, *I*_UC_, at 451 and 475 nm (black and red, respectively) is plotted against the output power of the NIR laser, *P*_exc_ (in Watt).


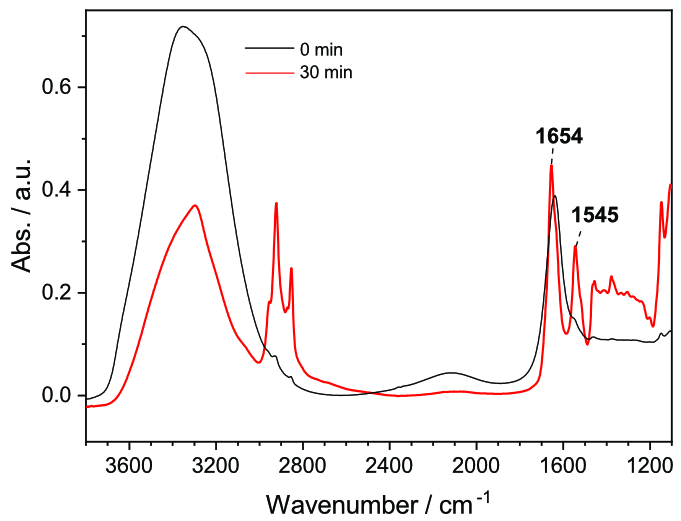


**Supplementary Figure 5.** FTIR spectrum of the *Np*SRII film after 0 min, black, and 30 min, red, of air-drying.
